# Low-Dose Curcumin Nanoparticles Normalise Blood Pressure in Male Wistar Rats with Diet-Induced Metabolic Syndrome

**DOI:** 10.3390/nu11071542

**Published:** 2019-07-08

**Authors:** Ryan du Preez, Jessica Pahl, Meenakshi Arora, M. N. V. Ravi Kumar, Lindsay Brown, Sunil K. Panchal

**Affiliations:** 1 Functional Foods Research Group, University of Southern Queensland, Toowoomba, QLD 4350, Australia; 2 School of Health and Wellbeing, University of Southern Queensland, Toowoomba, QLD 4350, Australia; 3 Texas A&M Rangel College of Pharmacy, Texas A&M University–College Station, TX 77843, USA

**Keywords:** metabolic syndrome, curcumin, nanoparticles, hypertension, obesity, oral bioavailability

## Abstract

Nanoparticle formulations improve bioavailability and so may allow low-dose formulations of food-derived compounds such as curcumin to attenuate chronic systemic disease despite intrinsically low oral bioavailability. The current study induced metabolic syndrome in male Wistar rats aged eight–nine weeks using a high-carbohydrate, high-fat diet (H) with corn starch diet (C) as control. Using a reversal protocol, rats were given curcumin as either nanoparticles encapsulated in poly(lactic–co–glycolic acid) (5 mg/kg/day, HCNP) or as an unformulated low dose or high-dose suspension in water (low-dose, 5 mg/kg/day, HC5; high-dose, 100 mg/kg/day, HC100) or blank nanoparticles (HBNP) for the final eight weeks of the 16 week study. We analysed cardiovascular parameters including systolic blood pressure and left ventricular diastolic stiffness along with histopathology, liver parameters including plasma liver enzymes, histopathology and metabolic parameters, including glucose tolerance, blood lipid profile and body composition, and plasma curcumin concentrations. HC100 and HCNP but not HBNP normalised systolic blood pressure (C = 120 ± 4; H = 143 ± 5; HBNP = 141 ± 3; HC5 = 143 ± 4; HC100 = 126 ± 4; HCNP = 128 ± 4 mmHg), left ventricular diastolic stiffness and liver fat deposition. No other improvements were induced in HC100 or HCNP or other intervention groups (HC5 and HBNP). We conclude that 5 mg/kg/day curcumin nanoparticles in H rats showed similar improvements in cardiovascular function as 100 mg/kg/day unformulated curcumin correlating with similar plasma curcumin concentrations.

## 1. Introduction

Curcumin is the major active constituent of turmeric, isolated from the rhizomes of *Curcuma longa*. Turmeric is commonly used as a spice and food-colouring agent while curcumin has been proposed for the management of a wide range of diseases including metabolic syndrome, arthritis, anxiety and hyperlipidaemia [1], as well as ageing-associated diseases including cardiovascular disease and chronic inflammation [2]. The broad-spectrum activity of curcumin has been attributed to its ability to modulate many transcription factors, cytokines, growth factors and enzymes [3]. However, the therapeutic usefulness of curcumin has been strongly questioned because of its interference with many assays and producing misleading and false results leading to the description of curcumin as an unstable, reactive and non-bioavailable compound, and to its classification as a pan-assay interference compound and an invalid metabolic panacea candidate [4]. This review concluded that curcumin does not warrant further investigation as a therapeutic agent [4]. This is in marked contrast with the many clinical trials that have shown safety, tolerability and effectiveness of curcumin against chronic diseases in humans [1,2,3,5].

Turmeric contains hundreds of potentially bioactive compounds with curcumin present at 0.4%–7.2% of dry weight [6]. However, curcumin has very low oral bioavailability in rats, due to its hydrophobic nature, rapid metabolism and systemic elimination. A comparison of intravenous and oral administration routes showed that 10 mg/kg intravenous curcumin produced an area under the curve of 7.2 ± 1.2 *μ*g/mL × minutes, in contrast to 3.6 ± 0.6 *μ*g/mL × minutes following 500 mg/kg oral curcumin, thus showing ~1% oral bioavailability [7]. Alternative delivery methods have focussed on increasing bioavailability and absorption, including adjuvants, liposomes, micronisation and nanoparticles, aiming to produce health benefits using a lower dose of curcumin [8]. Adjuvants such as piperine inhibited hepatic and intestinal glucuronide conjugation to increase curcumin bioavailability [9]. Liposomes should be an effective carrier to enhance the stability, bioavailability and targeting property of curcumin [10]. A *γ*-cyclodextrin curcumin formulation increased relative bioavailability of total curcuminoids in healthy humans by 39-fold compared to standard curcumin [11]. Nanoparticle preparations of curcumin encapsulated in poly (lactic–co–glycolic acid) (PLGA) increased bioavailability by ~9-fold more than co-administration with piperine which was 3-fold more than unformulated curcumin [12]. Curcumin nanoformulations increased solubilisation of curcumin and protected curcumin against inactivation by hydrolysis [13].

Metabolic syndrome is defined as a clustering of risk factors associated with increased risk of cardiovascular disease and diabetes, including abdominal obesity, hypertension, impaired glucose tolerance, insulin resistance, decreased high-density lipoprotein-cholesterol and elevated triglycerides [14]. Among US adults, metabolic syndrome has increased by more than 35% in the last two decades [15]. The use of functional foods and nutraceuticals could produce a reliable decrease in metabolic syndrome with decreased comorbidities. This outcome would be superior to current drug treatments [16].

In the current study, metabolic syndrome was induced in male rats by feeding a diet with increased simple sugars such as fructose and sucrose together with increased saturated and *trans* fats. These rats were treated with PLGA nanoparticle-encapsulated curcumin using a reversal protocol since these nanoparticles showed improved biocompatibility, biodegradability and capacity to prevent premature degradation of curcumin without toxicity during chronic dosing [17,18]. PLGA nanoparticle encapsulation of curcumin improved oral bioavailability, delayed cataract progression and prevented *ß*-cell death [12,17,19]; however, this is the first study investigating oral low-dose curcumin PLGA-nanoparticles in a rat model of human metabolic syndrome. PLGA has been used in products that are in human use and is a widely used polymer for drug delivery applications [20].

Curcumin was delivered orally to rats as a suspension at 5 or 100 mg/kg/day to provide both an equal dose and an approximately equal predicted absorption of curcumin at 100 mg/kg/day with PLGA nanoparticles at 5 mg/kg/day. After treatment with curcumin, the structure and function of the cardiovascular system and liver were examined. Cardiovascular structure and function were studied in isolated Langendorff heart preparations and by measurement of systolic blood pressure together with histopathology. Liver structure and function were measured by plasma biochemical analysis and histopathology. Metabolic parameters related to obesity and glucose tolerance were also evaluated. Further, we characterised the composition of the gut microbiome after curcumin treatment, since obesity-induced changes in the gut microbiome in metabolic disorders, including cardiovascular disease, may be reversed by functional foods, nutraceuticals and herbal medicines [21,22]. We hypothesised that rats given low-dose curcumin nanoparticles (5 mg/kg/day) will have similar chronic physiological responses as rats given the high-dose curcumin suspension (100 mg/kg/day) and much greater than rats given the low-dose curcumin suspension (5 mg/kg/day), showing that a lower curcumin dose in nanoparticles is effective against signs of metabolic syndrome.

## 2. Materials and Methods

### 2.1. Curcumin Suspension and Nanoparticles

Curcumin was obtained from Acros Organics (Morris Plains, NJ, USA) as a mixture of curcumin (>98%) with demethoxycurcumin and bisdemethoxycurcumin (both <1%). Curcumin aqueous suspensions were prepared as 5 and 100 mg/mL. PLGA ((50:50) Resomer R503H; MW 35-40kDa) was used to prepare curcumin nanoparticles (CNP) by single emulsion method resulting in particles with a hydrodynamic diameter of 315–320 nm [17,19]. Blank nanoparticles (BNP) without curcumin were used as the vehicle control and had a hydrodynamic diameter of 310 nm. Both CNP and BNP had surface charge measured as zeta potential of –29 mV at pH 5.5.

### 2.2. Rats and Diets

All experimental protocols were approved by the Animal Ethics Committee of the University of Southern Queensland (approval number 15REA008) under the guidelines of the National Health and Medical Research Council of Australia. Male Wistar rats (8–9 weeks old; 338 ± 1 g, *n* = 120) were obtained from the Animal Resource Centre, Murdoch, WA, Australia. Rats were individually housed in a temperature-controlled (21 ± 2 °C), 12-hour light/dark conditions with free access to food and water. Rats were randomly distributed into ten groups, each with 12 rats. For the first eight weeks, half the rats received a corn starch (C) diet and the other half received a high-carbohydrate, high-fat (H) diet [23]. Two groups (C and H rats) received the diet uninterrupted throughout the remaining eight weeks of the sixteen-week study. The remaining eight groups were administered curcumin suspension (5 or 100 mg/kg/day; CC5, CC100, HC5 or H100) or blank or curcumin nanoparticles (CBNP, CCNP, HBNP or HCNP) by once-daily oral gavage together with C or H diet for the remaining eight weeks. The energy densities were 11.23 kJ/g for the C food, 17.83 kJ/g for the H food with an additional 3.85 kJ/mL from the fructose in the drinking water of H rats.

### 2.3. Measurements Before Euthanasia

Systolic blood pressure was measured under light sedation with Zoletil (10 mg/kg tiletamine, 10 mg/kg zolazepam, i.p; Virbac, Peakhurst, NSW, Australia) [23]. Measurements were performed using an MLT1010 Piezo-Electric Pulse Transducer (ADInstruments, Bella Vista, NSW, Australia) and an inflatable tail-cuff connected to an MLT844 Physiological Pressure Transducer (ADInstruments) connected to a PowerLab data acquisition unit (ADInstruments).

Oral glucose tolerance tests were performed on rats after overnight (12-hour) food deprivation. Fructose-supplemented drinking water in H, HC5, HC100, HBNP and HCNP rats was replaced with normal drinking water. Basal blood glucose concentrations were determined in tail vein blood using Medisense Precision Q.I.D. glucometer (Abbott Laboratories, Bedford, MA, USA) and glucose test strips (Freestyle Optium Blood Glucose Test Strips, Abbott Diabetes Care Ltd, Milperra NSW, Australia). Rats were then given 2 g/kg body weight of glucose as a 40% (w/v) aqueous solution by oral gavage. Following this, blood glucose concentrations were measured at 30, 60, 90 and 120 min following glucose administration [23].

Dual-energy X-ray absorptiometry was performed on all rats after 16 weeks of feeding using a Norland XR46 DXA instrument (Norland Corp., Fort Atkinson, WI, USA). Rats were sedated with Zoletil (10 mg/kg tiletamine, 10 mg/kg zolazepam; i.p). Scans were analysed using the manufacturer’s recommended software for use in laboratory animals (Small Subject Analysis Software, version 2.5.3/1.3.1; Norland Corp.) [23].

Indirect calorimetry was used to measure oxygen consumption and carbon dioxide production using a 4-chamber Oxymax system (Columbus Instruments, Columbus, OH, USA) with one rat per chamber. Rats had free access to food and water during the measurement. Oxygen consumption (V_O2_) and carbon dioxide production (V_CO2_) were measured individually from each chamber. The respiratory exchange ratio (RER = V_CO2_/V_O2_) was calculated by Oxymax software (v. 4.86). The oxidation of carbohydrates produces an RER of 1.00, whereas fatty acid oxidation results in an RER of about 0.70. Energy expenditure (heat) was calculated by assessment of the exchange of oxygen for carbon dioxide that occurs during the metabolic processing of food [24].

### 2.4. Measurements After Euthanasia

Rats were euthanased via intraperitoneal injection of Lethabarb (pentobarbitone sodium, 100 mg/kg; Virbac). Once euthanasia was induced in rats, heparin was administered (~200 IU) into the right femoral vein. The abdomen was then opened and blood (~6 mL) was withdrawn from the abdominal aorta, collected into heparinised tubes and centrifuged at 5000 × *g* for 10 min. Plasma from each rat was stored at −20 °C before analysis. Hearts were removed for use in the isolated Langendorff heart preparation. Hearts were perfused with a modified Krebs-Henseleit bicarbonate buffer [23]. Buffer was bubbled with 95% O_2_–5% CO_2_ and maintained at 35 °C. Isovolumetric ventricular function was measured by inserting a latex balloon catheter into the left ventricle connected to a Capto SP844 MLT844 physiological pressure transducer and Chart software on a MacLab system. All left ventricular end-diastolic pressure values were measured during pacing of the heart at 250 beats/min using an electrical stimulator. End-diastolic pressures were obtained from 0 to 30 mmHg for calculation of diastolic stiffness constant (*κ*, dimensionless) [23].

After completing the Langendorff heart preparation, hearts were separated into right ventricle and left ventricle with septum for weighing. Livers and abdominal fat pads (retroperitoneal, epididymal and omental) were isolated and weighed. These organ weights were normalised relative to the tibial length at the time of their removal (in mg of tissue/mm of tibial length) [23].

Two rats from each group were exclusively used for histological analysis. Tissues were also collected from two other rats in each group. Approximately 5–7 min after euthanasia, heart, liver and intestinal tissues were collected and fixed in 10% neutral buffered formalin for 3 days. The samples were then dehydrated and embedded in paraffin wax. Two slides were prepared for each heart and liver specimen and two random, non-overlapping fields per slide were taken to avoid biased analysis. Thin sections (5 μm) of the samples were cut and stained with haematoxylin and eosin for determination of inflammatory cell infiltration (20×) and presence of liver fat vacuoles (40×) or picrosirius red stain for collagen deposition. EVOS FL Colour Imaging System (v1.4 (Rev 26059); Advanced Microscopy Group, Bothell, WA, USA) was used to capture images to determine the extent of collagen deposition in selected tissue sections [23]. NIH ImageJ software was used to quantify collagen deposition in heart sections and to count inflammatory cells in liver sections.

Plasma samples collected during terminal experiments were used to test for enzyme activities and plasma concentrations of biochemical markers. Plasma activities of alanine transaminase (ALT) and aspartate transaminase (AST) and plasma concentrations of total cholesterol, triglycerides and non-esterified fatty acids were determined using kits and controls [23].

Immediately following euthanasia and organ removal, two or three faecal pellets were collected from the colon of rats and stored at −80 °C in nuclease-free tubes. DNA extraction and diversity profiling were performed by the Australian Genome Research Facility, Brisbane, QLD, Australia. The V3-V4 region of the 16S rRNA gene was selected for amplification. The primers used were F341 (50-CCTAYGGGRBGCASCAG-30) and R806 (50-GGACTACNNGGGTATCTAAT-30). PCR amplicons were generated using AmpliTaq Gold 360 mastermix (Life Technologies, Scoresby, VIC, Australia) for the primary PCR. Detailed description for the analysis of diversity profiling is available in our previous study [25].

Plasma concentrations of curcumin were measured by LC-MS/MS. Stock solutions at a concentration of 1.0 mg/mL were prepared by separately dissolving 1 mg of curcumin, curcumin glucuronide and salbutamol (internal standard) in 1 mL of acetonitrile. Standard working solutions were then prepared by dilution of curcumin and curcumin glucuronide stock solutions with acetonitrile to obtain working solutions with concentrations of 1, 2.5, 5, 10, 25, 50, 100 and 250 µg/mL. Calibration standards were prepared by spiking 30 µL of blank rat serum with a freshly prepared working solution at concentrations of 1, 2.5, 5, 10, 25, 50, 100 and 250 µg/mL and extracting with 10 ng/mL salbutamol in 100% acetonitrile to achieve standards with concentrations of 0.033, 0.0833, 0.166, 0.33, 0.833, 1.66, 3.33 and 8.33 µg/mL. For sample preparation, 30 µL of rat plasma samples were placed in a 1.5 mL Eppendorf tube, 120 µL of ice-cold 10 ng/mL salbutamol in 100% acetonitrile was added and the mixture was vortexed. Samples were sonicated in a water bath for 1 min and then centrifuged at 15,000 rpm for 5 min. After centrifugation, 200 µL of the supernatant was transferred to a clean 1.5 mL Eppendorf tube. 50 µL of the supernatant was then transferred to a 2 mL glass vial with a glass insert from where 10 μL was injected into the LC–MS/MS system. The target compounds in samples were detected and quantified on a triple quadrupole mass spectrometer (Quantiva, Thermo Fisher Scientific, Waltham, MA, USA) coupled to a binary pump HPLC (UltiMate 3000, Thermo Fisher Scientific). MS parameters were optimised for the target compound under direct infusion at 5 µL/min to identify the SRM transitions (precursor/product fragment ion pair) with the highest intensity in positive mode as 369.4–177 m/z for curcumin, 545.2–369 m/z for curcumin glucuronide and 240.2–148.2 m/z for salbutamol. Samples were maintained at 4 °C on an autosampler before injection. The injection volume was 10 µL. Chromatographic separation was achieved on a Hypersil Gold 5 µm 50 × 3 mm column (Thermo Scientific) maintained at 30 °C using a solvent gradient method. Solvent A was water with 0.1% formic acid. Solvent B was acetonitrile with 0.1% formic acid. The gradient method used was 0–4 min (20% B to 80% B), 4–4.1 min (80% B to 95% B), 4.1–6 min (95% B), 6–6.5 min (95% B to 20% B) and 6.5–8 min (20% B). The flow rate was 0.5 mL/min. Sample acquisition and analysis were performed with TraceFinder 3.3 (Thermo Scientific).

### 2.5. Statistical Analysis

All data are presented as mean ± standard error of the mean (SEM). Results were tested for variance using Bartlett’s test and variables that were not normally distributed were transformed (using log10 function) prior to statistical analyses. Data from the ten treatment groups were tested by two-way analysis of variance. When the interactions and/or the main effects were significant, means were compared using the Newman-Keuls multiple comparison post hoc test. Where transformations did not result in normality or constant variance, a Kruskal-Wallis non-parametric test was performed. A *p* value of <0.05 was considered as statistically significant. All statistical analyses were performed using GraphPad Prism version 5.00 for Windows (GraphPad Software, San Diego, CA, USA).

## 3. Results

### 3.1. Body Parameters and Dietary Intakes

Rats fed a high-carbohydrate, high-fat diet as simple sugars and both saturated and *trans* fats (H rats) developed symptoms characteristic of human metabolic syndrome, including abdominal obesity (shown as increases in body weight, fat mass and abdominal fat pads), hypertension (shown as increases in systolic blood pressure), dyslipidaemia (shown as increases in plasma triglycerides and non-esterified fatty acids) and impaired glucose tolerance (shown as increases in 120-min blood glucose concentrations and area under the curve) compared to rats fed the corn starch diet (C rats) (Table 1). Body weights were unchanged with the interventions compared to their respective controls (Table 1). Lean mass was unchanged in all groups (Table 1). Fat mass was unchanged between C groups or between H, HC5, HC100 and HCNP rats (Table 1). Food intake was higher in C rats compared to H rats. CC5 and CC100 rats had similar food intake to C rats, whereas CCNP had higher food intake than CBNP rats, which were higher than C, CC5 and CC100 rats (Table 1). With lower body weight gain, all C diet-fed groups had lower feed efficiency compared to H diet-fed groups (Table 1). Plasma curcumin concentrations measured at the end of the protocol were unchanged between CC100 and CCNP or HC100 and HCNP groups, suggesting improved oral bioavailability with nanoparticle delivery. On the other hand, plasma curcumin concentrations in CC100 was higher than in HC100 rats, but not in CCNP and HCNP rats. Curcumin was not detected in the plasma from HC5 rats (Table 1).

### 3.2. Metabolic Changes

There were no differences between C and H rats in heat produced and RER (Table 1). CCNP rats had lower heat production than C rats, while HCNP and HBNP rats had lower heat production than H rats (Table 1). Total abdominal fat was higher in HBNP rats compared to all other groups. H, HC5, HC100 and HCNP rats had similar fat weight, which was higher than all C diet-fed groups (Table 1). Plasma triglyceride concentrations were higher in H diet-fed groups compared to C diet-fed groups. Plasma total cholesterol concentrations were highest in HBNP group and lower in CC5, CC100, H and HC100 rats, while other groups were intermediate (Table 1). Plasma non-esterified fatty acids were higher in H diet-fed rats compared to C diet-fed rats. C and H rats had similar basal blood glucose concentrations. None of the interventions reduced basal blood glucose concentrations (Table 1). The blood glucose area under the curve was not different between H diet-fed rats (Table 1).

### 3.3. Cardiovascular Changes

**Figure 1:**
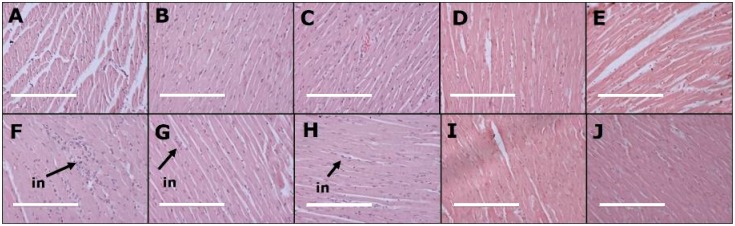
Effects of curcumin on inflammatory cells (“in”) in the heart using haematoxylin and eosin stain in corn starch diet-fed rats (**A**), corn starch diet-fed rats given 5 mg/kg/day curcumin (**B**), corn starch diet-fed rats given 100 mg/kg/day curcumin (**C**), corn starch diet-fed rats given 5 mg/kg/day curcumin nanoparticles (**D**), corn starch diet-fed rats given blank nanoparticles (**E**), high-carbohydrate, high-fat diet-fed rats (**F**), high-carbohydrate, high-fat diet-fed rats given 5 mg/kg/day curcumin (**G**), high-carbohydrate, high-fat diet-fed rats given 100 mg/kg/day curcumin (**H**), high-carbohydrate, high-fat diet-fed rats given 5 mg/kg/day curcumin nanoparticles (**I**) and high-carbohydrate, high-fat diet-fed rats given blank nanoparticles (**J**). The white bar is 200 μm.

**Figure 2:**
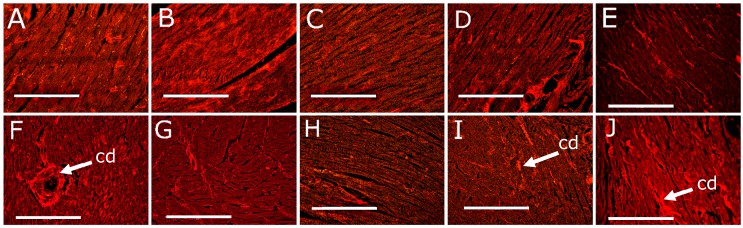
Effects of curcumin on collagen deposition (“cd”) in the heart using picrosirius red stain in corn starch diet-fed rats (**A**), corn starch diet-fed rats given 5 mg/kg/day curcumin (**B**), corn starch diet-fed rats given 100 mg/kg/day curcumin (**C**), corn starch diet-fed rats given 5 mg/kg/day curcumin nanoparticles (**D**), corn starch diet-fed rats given blank nanoparticles (**E**), high-carbohydrate, high-fat diet-fed rats (**F**), high-carbohydrate, high-fat diet-fed rats given 5 mg/kg/day curcumin (**G**), high-carbohydrate, high-fat diet-fed rats given 100 mg/kg/day curcumin (**H**), high-carbohydrate, high-fat diet-fed rats given 5 mg/kg/day curcumin nanoparticles (**I**) and high-carbohydrate, high-fat diet-fed rats given blank nanoparticles (**J**). The white bar is 200 μm.

After eight weeks, systolic blood pressure of H diet-fed groups (H, HC5, HC100, HCNP and HBNP) was higher than C diet-fed groups (C, CC5, CC100, CCNP and CBNP) (Table 1). After 16 weeks, systolic blood pressure in H rats was higher than in C rats. None of the interventions affected systolic blood pressure in C diet-fed groups. HC100 and HCNP had normalised systolic blood pressure, whereas HC5 and HBNP had no change in systolic blood pressure compared to H rats (Table 1). Diastolic stiffness was higher in H rats compared to C rats. HC100 and HCNP showed normalised diastolic stiffness, whereas other intervention groups did not change compared to their respective controls (Table 1). Left ventricular weights with septum were unchanged among all groups whereas right ventricular wet weights were lower in CC5, CCNP and HCNP groups (Table 1). Left ventricles from H rats showed infiltration of inflammatory cells (Figure 1F) and collagen deposition (Figure 2F) whereas these changes were not seen in left ventricles from C rats (Figure 1A and 2A; Table 1). Left ventricles from HCNP (Figure 1I) and HBNP (Figure 1J) rats showed decreased infiltration of inflammatory cells, whereas HC5 (Figure 2G), HC100 (Figure 2H) and HCNP (Figure 2I) showed decreased collagen deposition compared to H rats (Figure 1F and Figure 2F).

### 3.4. Liver Changes

**Figure 3:**
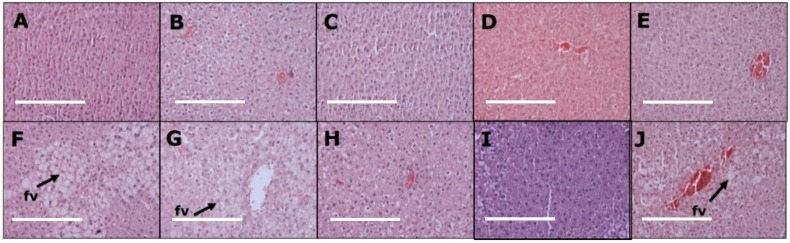
Effects of curcumin on fat deposition (“fv”) in the liver using haematoxylin and eosin stain in corn starch diet-fed rats (**A**), corn starch diet-fed rats given 5 mg/kg/day curcumin (**B**), corn starch diet-fed rats given 100 mg/kg/day curcumin (**C**), corn starch diet-fed rats given 5 mg/kg/day curcumin nanoparticles (**D**), corn starch diet-fed rats given blank nanoparticles (**E**), high-carbohydrate, high-fat diet-fed rats (**F**), high-carbohydrate, high-fat diet-fed rats given 5 mg/kg/day curcumin (**G**), high-carbohydrate, high-fat diet-fed rats given 100 mg/kg/day curcumin (**H**), high-carbohydrate, high-fat diet-fed rats given 5 mg/kg/day curcumin nanoparticles (**I**) and high-carbohydrate, high-fat diet-fed rats given blank nanoparticles (**J**). The white bar is 200 μm.

Livers from H rats (Figure 3F) showed increased fat deposition and infiltration of inflammatory cells compared to livers from C rats (Figure 3A). HC100 (Figure 3H) and HCNP (Figure 3I) rats had reduced fat deposition compared to H (Figure 3F), HC5 (Figure 3G) and HBNP rats (Figure 3J). Plasma activities of ALT and AST were not different between the groups (Table 1).

### 3.5. Gut Structure and Microbiome

**Figure 4:**
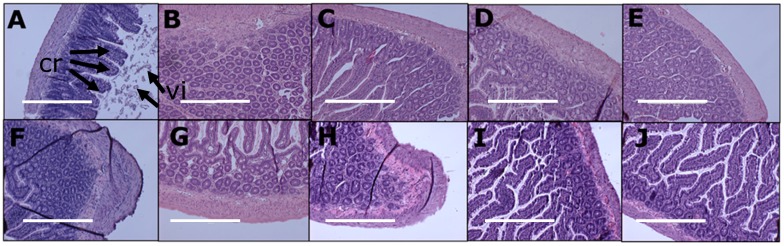
Effects of curcumin on structure, crypt depth (“cr”) and villi length (“vi”) in the ileum using haematoxylin and eosin stain in corn starch diet-fed rats (**A**), corn starch diet-fed rats given 5 mg/kg/day curcumin (**B**), corn starch diet-fed rats given 100 mg/kg/day curcumin (**C**), corn starch diet-fed rats given 5 mg/kg/day curcumin nanoparticles (**D**), corn starch diet-fed rats given blank nanoparticles (**E**), high-carbohydrate, high-fat diet-fed rats (**F**), high-carbohydrate, high-fat diet-fed rats given 5 mg/kg/day curcumin (G), high-carbohydrate, high-fat diet-fed rats given 100 mg/kg/day curcumin (**H**), high-carbohydrate, high-fat diet-fed rats given 5 mg/kg/day curcumin nanoparticles (**I**) and high-carbohydrate, high-fat diet-fed rats given blank nanoparticles (**J**). The white bar is 200 μm.

**Figure 5:**
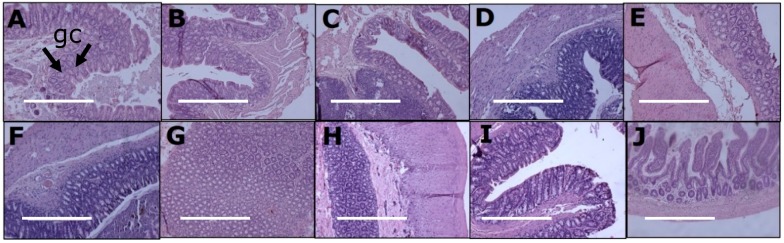
Effects of curcumin on structure and goblet cells (“gc”) in the colon using haematoxylin and eosin stain in corn starch diet-fed rats (**A**), corn starch diet-fed rats given 5 mg/kg/day curcumin (**B**), corn starch diet-fed rats given 100 mg/kg/day curcumin (**C**), corn starch diet-fed rats given 5 mg/kg/day curcumin nanoparticles (**D**), corn starch diet-fed rats given blank nanoparticles (**E**), high-carbohydrate, high-fat diet-fed rats (**F**), high-carbohydrate, high-fat diet-fed rats given 5 mg/kg/day curcumin (**G**), high-carbohydrate, high-fat diet-fed rats given 100 mg/kg/day curcumin (**H**), high-carbohydrate, high-fat diet-fed rats given 5 mg/kg/day curcumin nanoparticles (**I**) and high-carbohydrate, high-fat diet-fed rats given blank nanoparticles (**J**). The white bar is 200 μm.

Histology of ileum and colon did not show any abnormalities in the experimental groups demonstrated by normal crypt depth, villi length, goblet cells and lack of inflammatory cell infiltration (Figure 4 and Figure 5).

**Figure 6:**
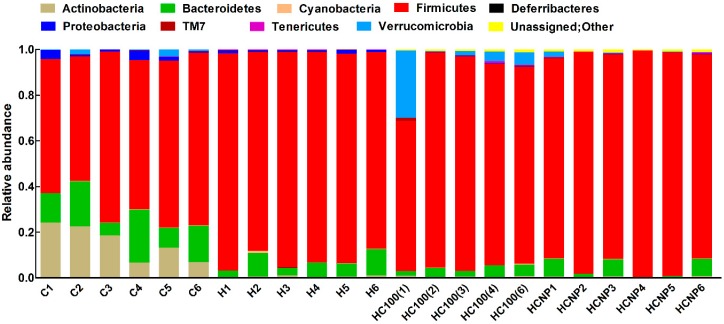
Effects of curcumin on gut microbiota at phylum level. C, corn starch diet-fed rats; H, high-carbohydrate, high-fat diet-fed rats; HC100, high-carbohydrate, high-fat diet-fed rats given 100 mg/kg/day curcumin; HCNP, high-carbohydrate, high-fat diet-fed rats given 5 mg/kg/day curcumin nanoparticles.

**Figure 7:**
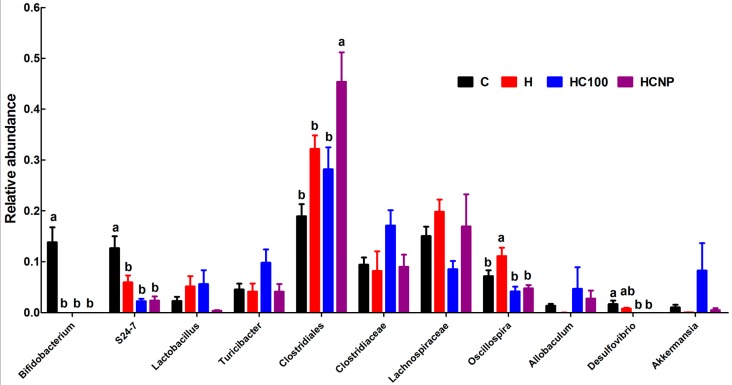
Effects of curcumin on gut microbiota at genus level. C, corn starch diet-fed rats; H, high-carbohydrate, high-fat diet-fed rats; HC100, high-carbohydrate, high-fat diet-fed rats given 100 mg/kg/day curcumin; HCNP, high-carbohydrate, high-fat diet-fed rats given 5 mg/kg/day curcumin nanoparticles.

In contrast to the histological findings, the high-carbohydrate, high-fat diet changed the colonic bacteria with increases in relative abundance of *Firmicutes* and decreases in relative abundance of *Bacteroidetes* in H rats compared to C rats (Figure 6). Moreover, H rats showed disappearance of *Actinobacteria* compared to C rats (Figure 6). Intervention with 100 mg/kg/day curcumin or curcumin nanoparticles did not change relative abundance of microbiota when compared to H rats (Figure 7).

## 4. Discussion

Both inflammation and oxidative stress are underlying causes of metabolic syndrome leading to an increased risk of developing cardiovascular disease and type 2 diabetes [14]. It has therefore been suggested that compounds such as curcumin may attenuate this syndrome [1], since preclinical studies [26] and clinical trials [27,28] have shown that curcumin has potent anti-inflammatory and anti-oxidative properties, although this is disputed [4]. This study shows that curcumin improved cardiovascular structure and function, especially with the normalisation of systolic blood pressure, left ventricular stiffness and left ventricular collagen deposition, in rats with diet-induced metabolic syndrome. Further, liver fat deposition was reduced. These cardiovascular and liver changes were comparable with high-dose curcumin suspension and low-dose curcumin nanoparticles.

Absorption and bioavailability of hydrophobic compounds such as curcumin can be improved by inclusion in high molecular weight (40–70 kDa) PLGA compared to low molecular weight (5–15 kDa) PLGA [29]. This current study used PLGA (35–40 kDa) to improve the absorption and bioavailability of curcumin. Products containing PLGA are approved by the Food and Drug Administration for human use as PLGA has superior biocompatibility and biodegradable properties [30]. PLGA copolymers are degraded in the body by hydrolytic cleavage of the ester linkages to lactic and glycolic acids. These compounds are easily metabolised in the body by the Krebs cycle and eliminated as carbon dioxide and water [30]. The PLGA nanoparticles used in the current study had a nine-fold improvement in oral bioavailability; they delivered a safe and potentially translatable dose of curcumin, which improved *β*-cell function [17]. In this study, curcumin PLGA nanoparticles at 5 mg/kg/day produced similar improvements in systolic blood pressure, left ventricular function and liver fat deposition as curcumin aqueous suspension at 100 mg/kg/day; however, both treatments caused minimal changes in metabolic parameters in high-carbohydrate, high-fat diet-fed rats. These physiological responses were accompanied by increased plasma concentrations of curcumin using PLGA nanoparticles even with a 20-fold lower dose.

Reduction in blood pressure by higher doses of curcumin and nanoparticles containing lower doses of curcumin could potentially be the reason for the reduced cardiac insult and hence improved ventricular inflammation and fibrosis. Possible mechanisms for these changes include modulation of vascular tone [31] and altering the predisposition to vascular disease by targeting Uncoupling Protein (UCP) 2 [32]. In middle-aged and older patients, curcumin improved resistance artery endothelial function by increasing nitric oxide bioavailability and reducing oxidative stress [33].

A recent systematic review found that curcumin improved anthropometric measurements in clinical trials in human adults; however, duration of study and dose/formulation are critical [34]. For example, feeding 1.6 g of curcuminoids daily for three months did not change total body fat and visceral fat, but after six months, both had decreased [35]. Curcumin intervention in mice inhibited high-fat diet-induced inflammation in white adipose tissue and activated UCP1 production in brown adipose tissue [36]. The decrease of inflammation in white adipose tissue and increase of energy expenditure through activation of brown adipose tissue are two key approaches to reduce obesity. Curcumin also prevented non-alcoholic steatohepatitis in rats [37]. In this study, we observed reduction in the fat deposition in liver, which is induced by high-carbohydrate, high-fat diet feeding [23]. In mice, a highly dispersible, low-dose curcumin formulation induced brown-like adipocyte formation more effectively than native curcumin [38]. This finding suggests curcumin formulations have effects *in vivo* at low doses, especially through increased energy expenditure. Curcumin reduced obesity and improved glucose sensitivity in rodents but these results have not been validated in human clinical trials [39]. Furthermore, the conflicting results require future trials that are randomised, double-blind and placebo-controlled to enable comparisons between studies [40]. A recent study concluded that the poor quality of the primary trials means that well-designed and long-term trials, using specific curcumin formulations, are required to make definitive conclusions regarding the efficacy of curcumin [41].

Curcumin (20 *μ*mol/L) induced browning of 3T3-L1 and primary adipocytes through inhibition of lipogenesis [42]. Further, intervention with curcumin (500 mg/kg diet for 12 weeks) reduced body weight in high-fat diet-fed mice while not affecting food intake [43]. These results contrast to the current study where there was no change in body weight or abdominal fat mass at lower doses of 5 or 100 mg/kg/day in rats for a shorter period of eight weeks. High-carbohydrate, high-fat diet-fed rats developed impaired glucose tolerance and hyperinsulinaemia [23]. In this study, there were no effects on glucose metabolism during oral glucose tolerance testing. As there were no changes in glucose responses in oral glucose tolerance testing, insulin concentrations and insulin sensitivity were not expected to change.

In a mouse model of colitis-associated colon cancer, curcumin reduced relative abundance of *Clostridiales* and increased the abundance of *Lactobacillales*, *Bifidobacteriales*, *Erisipelotrichales*, *Coriobacteriales* and the putative order YS2 from the Cyanobacteria phylum [44]. Other studies have identified modulation of gut microbiome by curcumin [45,46]. Gut microbiome has been reported to be a contributing factor in the development of obesity and obesity-related comorbidities [47]. Modulation of gut microbiome by functional foods such as prebiotics, probiotics and polyphenols has contributed to health benefits [48]. It has also been hypothesised that gastrointestinal effects of curcumin may be able to attenuate intestinal and extra-intestinal diseases [49]. Curcumin reduced local inflammation in the gut by altering intestinal barrier function and modulating the release of LPS into circulation suggesting that curcumin, despite its low oral bioavailability, may be able to mediate its effects through local actions in the gut [50]. Similar results were reported in another study where Western diet-induced changes in intestinal barrier function leading to increased endotoxaemia, macrophage activation and subsequent development of glucose intolerance and atherosclerosis were attenuated by curcumin [51]. In this study, curcumin and curcumin nanoparticles were unable to reverse any changes in gut microbiome induced by high-carbohydrate, high-fat diet, possibly because of the lower doses in this study. *Clostridiales* was increased by the curcumin nanoparticles, whereas *Oscillospira* was decreased by curcumin and curcumin nanoparticles.

This preliminary study with curcumin nanoparticles provided useful information in terms of improvement of blood pressure, ventricular hypertrophy, inflammation and fibrosis. The dose of curcumin through nanoparticles is 5 mg/kg/day for rats, which translates to 50 and 90 mg/day for an adult human using the body surface area and scaling equations, respectively [52,53]. The positive responses of low dose curcumin intervention through nanoparticles provides an avenue to test higher doses to potentially identify the potential metabolic effects of curcumin in diet-induced obesity. These future studies with higher doses of curcumin nanoparticles may warrant clinical evaluation in randomised clinical trials to identify the potential of this naturally occurring molecule with low bioavailability. Future studies may also include evaluation of markers such as specific types of inflammatory cells in the tissues causing damage in the heart and liver through immunohistochemistry to understand the selective roles of curcumin in inhibiting inflammation. Further, future studies may also include tissue evaluation of tissue markers of metabolic improvements such as the measurement of glycogen in the liver and muscles and activities of digestive and metabolic enzymes in the gut and liver.

## 5. Conclusions

PLGA-curcumin nanoparticles increased plasma curcumin concentrations and effectively improved cardiovascular responses and reduced liver fat deposition to similar extents at 20-fold lower doses of curcumin (5 mg/kg/day compared to 100 mg/kg/day), implying an improved oral bioavailability. The results indicate that a dose-response study with curcumin nanoparticles would help in determining the potential benefits of curcumin in metabolic diseases as usual doses would achieve much higher plasma concentrations than a capsule or an aqueous suspension.

## Figures and Tables

**Table 1:** Responses to curcumin and curcumin nanoparticles.

*Variables*	*C*	*CC5*	*CC100*	*CCNP*	*CBNP*	*H*	*HC5*	*HC100*	*HCNP*	*HBNP*
Initial body weight, g	337 ± 1	338 ± 1	338 ± 1	338 ± 1	339 ± 1	338 ± 1	339 ± 1	337 ± 1	337 ± 1	339 ± 1
Final body weight, g	393 ± 7 ^c^	388 ± 8 ^c^	380 ± 8 ^c^	403 ± 6 ^c^	380 ± 7 ^c^	514 ± 10 ^ab^	492 ± 9 ^b^	498 ± 9 ^b^	490 ± 6 ^b^	538 ± 16 ^a^
Body weight gain (weeks 9–16), %	9.7 ± 1.0 ^b^	9.0 ± 1.4 ^b^	9.6 ± 0.9 ^b^	11.0 ± 1.1 ^b^	8.9 ± 1.0 ^b^	23.4 ± 1.0 ^a^	19.4 ± 1.3 ^a^	19.8 ± 1.5 ^a^	21.6 ± 1.3 ^a^	21.7 ± 1.7 ^a^
Final lean mass, g	295 ± 5	289 ± 7	291 ± 8	288 ± 7	286 ± 8	320 ± 8	289 ± 6	291 ± 7	309 ± 11	303 ± 6
Final fat mass, g	85 ± 7 ^c^	70 ± 7 ^c^	66 ± 6 ^c^	104 ± 4 ^c^	77 ± 7 ^c^	184 ± 10 ^b^	185 ± 14 ^b^	202 ± 13 ^b^	169 ± 9 ^b^	233 ± 14 ^a^
Water intake (weeks 9–16), mL/day	31.9 ± 2.8	24.9 ± 1.2	28.5 ± 3.2	26.9 ± 1.6	30.6 ± 1.8	27.8 ± 1.1	31.6 ± 1.9	26.2 ± 1.2	31.3 ± 0.8	28.5 ± 1.5
Food intake (weeks 9–16), g/day	39.1 ± 1.3 ^c^	39.1 ± 2.2 ^c^	38.5 ± 1.5 ^c^	49.7 ± 0.7 ^a^	44.8 ± 2.1 ^b^	27.9 ± 0.9 ^ef^	23.7 ± 0.7 ^f^	25.0 ± 0.9 ^f^	33.6 ± 1.3 ^d^	31.3 ± 1.9 ^de^
Energy intake (weeks 9–16), kJ/day	439 ± 14 ^c^	432 ± 17 ^c^	446 ± 15 ^c^	559 ± 8 ^b^	512 ± 27 ^b^	588 ± 15^b^	561 ± 15 ^b^	539 ± 14 ^b^	714 ± 23 ^a^	661 ± 37 ^a^
Feed efficiency (weeks 9–16), g/kJ	0.07 ± 0.01 ^b^	0.07 ± 0.01 ^b^	0.07 ± 0.01 ^b^	0.07 ± 0.01 ^b^	0.06 ± 0.01 ^b^	0.15 ± 0.01 ^a^	0.13 ± 0.01 ^a^	0.15 ± 0.01 ^a^	0.12 ± 0.01 ^a^	0.15 ± 0.01 ^a^
Retroperitoneal fat, mg/mm	242 ± 13 ^d^	208 ± 15 ^d^	211 ± 10 ^d^	229 ± 13 ^d^	179 ± 17 ^d^	481 ± 27 ^b^	337 ± 37 ^c^	436 ± 22 ^b^	467 ± 22 ^b^	581 ± 39 ^a^
Epididymal fat, mg/mm	76 ± 8 ^cd^	79 ± 8 ^cd^	68 ± 8 ^d^	68 ± 8 ^d^	77 ± 6 ^cd^	171 ± 13 ^b^	171 ± 17 ^b^	143 ± 13 ^b^	126 ± 11 ^bc^	244 ± 29 ^a^
Omental fat, mg/mm	137 ± 10 ^c^	137 ± 12 ^c^	123 ± 7 ^c^	134 ± 7 ^c^	161 ± 20 ^c^	244 ± 14 ^ab^	224 ± 11 ^b^	219 ± 11 ^b^	219 ± 13 ^b^	278 ± 19 ^a^
Total abdominal fat, mg/mm	455 ± 25 ^c^	423 ± 34 ^c^	402 ± 23 ^c^	432 ± 24 ^c^	417 ± 38 ^c^	895 ± 44 ^b^	827 ± 51 ^b^	797 ± 39 ^b^	813 ± 38 ^b^	1103 ± 70 ^a^
Left ventricle + septum weight, mg/mm	23.8 ± 1.4	21.9 ± 0.7	21.0 ± 0.8	22.7 ± 1.1	19.9 ± 0.8	23.4 ± 0.8	22.5 ± 0.5	23.4 ± 1.0	23.4 ± 0.8	24.2 ± 1.0
Right ventricular weight, mg/mm	5.1 ± 0.3 ^abc^	4.1 ± 0.3 ^cd^	4.7 ± 0.3 ^bc^	3.4 ± 0.3 ^d^	4.1 ± 0.5 ^cd^	5.7 ± 0.2 ^ab^	4.8 ± 0.3 ^abc^	4.9 ± 0.2 ^abc^	4.0 ± 0.2 ^cd^	5.9 ± 0.1 ^a^
Metabolic variables										
Heat, kcal	3.87 ± 0.08 ^ab^	3.86 ± 0.09 ^ab^	3.48 ± 0.41 ^b^	2.70 ± 0.18 ^c^	3.39 ± 0.26 ^b^	4.34 ± 0.09 ^a^	4.22± 0.12 ^a^	4.28 ± 0.10 ^a^	3.26 ± 0.21 ^b^	3.45 ± 0.13 ^b^
RER	1.03 ± 0.03 ^ab^	1.03 ± 0.10 ^ab^	1.04 ± 0.02 ^a^	1.03 ± 0.02 ^ab^	1.02 ± 0.02 ^ab^	0.91 ± 0.01 ^ab^	0.92 ± 0.01 ^ab^	0.90 ± 0.01 ^ab^	0.87 ± 0.02 ^b^	0.92 ± 0.01 ^ab^
Plasma triglycerides, mmol/L	0.53 ± 0.06 ^b^	0.49 ± 0.07 ^b^	0.41 ± 0.03 ^b^	0.59 ± 0.06 ^b^	0.83 ± 0.15 ^b^	1.71 ± 0.45 ^a^	1.77 ± 0.56 ^a^	1.53 ± 0.06 ^a^	1.52 ± 0.15 ^a^	1.64 ± 0.21 ^a^
Plasma total cholesterol, mmol/L	1.64 ± 0.08 ^ab^	1.45 ± 0.06 ^b^	1.44 ± 0.06 ^b^	1.60 ± 0.06 ^ab^	1.73 ± 0.13 ^ab^	1.53 ± 0.08 ^b^	1.71 ± 0.10 ^ab^	1.49 ± 0.09 ^b^	1.74 ± 0.05 ^ab^	1.93 ± 0.13 ^a^
Plasma non-esterified fatty acids, mmol/L	1.40 ± 0.20 ^cd^	1.28 ± 0.09 ^cd^	0.96 ± 0.08 ^d^	1.58 ± 0.16 ^cd^	2.42 ± 0.33 ^bc^	3.30 ± 0.40 ^ab^	2.64 ± 0.68 ^bc^	4.03 ± 0.36 ^a^	3.73 ± 0.18 ^ab^	4.50 ± 0.63 ^a^
Basal blood glucose, mmol/L	3.2 ± 0.1 ^c^	3.6 ± 0.1 ^bc^	3.2 ± 0.1 ^c^	3.9 ± 0.1 ^abc^	3.3 ± 0.2 ^c^	3.4 ± 0.2 ^bc^	3.9 ± 0.2 ^abc^	3.9 ± 0.4 ^abc^	4.5 ± 0.2 ^a^	4.2 ± 0.2 ^ab^
120-min blood glucose, mmol/L	4.6 ± 0.4 ^bcd^	4.2 ± 0.1 ^cd^	3.7 ± 0.1 ^d^	4.5 ± 0.2 ^bcd^	3.6 ± 0.2 ^d^	6.0 ± 0.6 ^a^	5.1 ± 0.2 ^abc^	5.4 ± 0.3 ^abc^	4.3 ± 0.2 ^cd^	5.6 ± 0.4 ^ab^
Blood glucose area under the curve, mmol/L×min	665 ± 8 ^ab^	591 ± 11 ^bc^	561 ± 18 ^c^	664 ± 14 ^ab^	560 ± 22 ^c^	739 ± 35 ^a^	695 ± 15 ^a^	673 ± 28 ^ab^	705 ± 14 ^a^	692 ± 42 ^a^
Plasma ALT activity, U/L	36 ± 2	41 ± 4	40 ± 5	32 ± 3	32 ± 3	42 ± 2	47 ± 5	40 ± 3	41 ± 3	42 ± 4
Plasma AST activity, U/L	88 ± 2	103 ± 6	90 ± 5	83 ± 3	87 ± 4	95 ± 2	105 ± 10	99 ± 9	88 ± 4	97 ± 10
Plasma curcumin concentrations, ng/ml	-	97.4 ± 18.0 ^b^	337.7 ± 84.7 ^a^	199.7 ± 45.3 ^b^	-	-	0.0 ± 0.0 ^c^	146.0 ± 21.2 ^b^	110.7 ± 17.8 ^b^	-
Liver inflammatory cells (cells/200µm^2^)	5 ± 1 ^c^	6 ± 2 ^c^	5 ± 1 ^c^	5 ± 2 ^c^	5 ± 2 ^c^	23 ± 2 ^a^	15 ± 3 ^b^	16 ± 2 ^b^	15 ± 1 ^b^	16 ± 1 ^b^
Cardiovascular variables										
16 week systolic blood pressure, mmHg	120 ± 4 ^b^	125 ± 1 ^b^	122 ± 2 ^b^	125 ± 2 ^b^	130 ± 3 ^b^	143 ± 5 ^a^	143 ± 4 ^a^	126 ± 4 ^b^	128 ± 4 ^b^	141 ± 3 ^a^
Left ventricular diastolic stiffness constant (κ)	22.0 ± 0.8 ^b^	21.8 ± 0.6 ^b^	23.3 ± 0.9 ^b^	22.8 ± 0.8 ^b^	21.9 ± 0.5 ^b^	28.9 ± 0.8 ^a^	28.5 ± 0.7 ^a^	23.4 ± 1.1 ^b^	23.5 ± 0.7 ^b^	27.9 ± 0.9 ^a^
Left ventricle collagen area, %	11 ± 1 ^c^	13 ± 1 ^c^	12 ± 1 ^c^	15 ± 1 ^c^	16 ± 1 ^c^	38 ± 2 ^a^	22 ± 2 ^b^	24 ± 2 ^b^	22 ± 2 ^b^	27 ± 1 ^b^

Values are presented as mean ± SEM, *n* = 10–12 (*n* = 4 for plasma curcumin). Means in a row with unlike superscripts (a, b, c, d, e or f) differ, *p* < 0.05. ALT, alanine transaminase; AST, aspartate transaminase; C, corn starch diet-fed rats; CC5, corn starch diet-fed rats given 5 mg/kg/day curcumin; CC100, corn starch diet-fed rats given 100 mg/kg/day curcumin; CCNP, corn starch diet-fed rats given 5 mg/kg/day curcumin nanoparticles; CBNP, corn starch diet-fed rats given blank nanoparticles; H, high-carbohydrate, high-fat diet-fed rats; HC5, high-carbohydrate, high-fat diet-fed rats given 5 mg/kg/day curcumin; HC100, high-carbohydrate, high-fat diet-fed rats given 100 mg/kg/day curcumin; HCNP, high-carbohydrate, high-fat diet-fed rats given 5 mg/kg/day curcumin nanoparticles; HBNP, high-carbohydrate, high-fat diet-fed rats given blank nanoparticles; RER, respiratory exchange ratio.
